# Chemically-induced cellular stress signals are transmitted to alternative splicing via UsnRNA levels to alter gene expression in *Arabidopsis thaliana*

**DOI:** 10.1007/s11103-025-01575-9

**Published:** 2025-03-16

**Authors:** Hirokazu Takahashi, Toshihiro Arae, Kodai Ishibashi, Ryosuke Sano, Taku Demura, Misato Ohtani

**Affiliations:** 1 Graduate School of Science and Technology, Nara Institute of Science and Technology, Ikoma, Nara, 630-0192 Japan; 2 RIKEN Center for Sustainable Resource Science, 1-7-22 Suehiro-Cho, Tsurumi-Ku, Yokohama, 230-0045 Japan; 3 Department of Integrated Biosciences, Graduate School of Frontier Sciences, The University of Tokyo, 5-1-5 Kashiwanoha, Kashiwa, 277-8562 Japan

**Keywords:** Alternative splicing, *Arabidopsis thaliana*, Cellular stress inducer, SRD2, UsnRNA

## Abstract

Alternative pre-mRNA splicing (AS) is a crucial regulatory layer of gene expression in eukaryotes. AS patterns can change in response to abiotic and biotic stress, allowing cellular functions to adapt to environmental conditions. Here, we examined the effects of cellular stress-inducing chemicals on AS-mediated gene regulation in *Arabidopsis thaliana* by investigating the alternatively spliced forms of *SERINE-ARGININE PROTEIN30* (*SRp30*) and *U1-70 K*, encoding splicing factors, as well as *ASCORBATE PEROXIDASE3* (*APX3*) and *FOLYLPOLYGLUTAMATE SYNTHASE3* (*FPGS3*), encoding enzymes important for stress responses. Disrupting key cellular activities, including nitric oxide metabolism, ATPase activity, plastid function, and genome stability, affected AS patterns in *Arabidopsis*. Stress treatment altered the abundance of uridine-rich small nuclear RNAs (UsnRNAs), especially U1 snRNAs, which are essential non-coding RNA components of U1 small nuclear ribonucleoproteins (U1 snRNPs), suggesting that abnormalities in AS are partially mediated by changes in U1 snRNA levels. The *shoot redifferentiation defectice2-1* (*srd2-1*) mutant defective for snRNA transcription was hypersensitive for stress treatment, since it showed changes in AS patterns at lower concentrations of stress inducers to compare with the wild type. Together, our data suggest that cellular stress can influence gene expression in plants by regulating AS, which is partially regulated by UsnRNA levels through the SRD2-mediated snRNA transcription.

**Supplementary Information:**

The online version contains supplementary material available at 10.1007/s11103-025-01575-9.

## Key message

In plants, cellular stress differentially changes gene expression by regulating alternative splicing, which is regulated by UsnRNA levels through SRD2-mediated transcription.

**Supplementary Information:**

The online version contains supplementary material available at 10.1007/s11103-025-01575-9.

## Introduction

In eukaryotic cells, most mRNA molecules are generated as pre-mRNAs containing intron sequences, which are removed during pre-mRNA splicing to produce mature mRNAs. Pre-mRNA splicing is performed by the spliceosome, a huge RNA–protein complex containing small nuclear ribonucleoproteins (snRNPs) composed of uridine-rich small nuclear RNAs (UsnRNAs) and their specific interacting proteins, as well as accessory proteins (Lorković et al. 2000). Due to the essential role of the spliceosome in producing mature mRNA, the regulation of spliceosome function is critical for cellular activities in eukaryotes. Loss-of-function mutations of various spliceosome-related genes are frequently lethal in animals, fungi, and plants (Tsukaya et al. 2013). For example, knockout of the gene encoding the UsnRNA-specific transcription factor SHOOT REDIFFERENTIATION DEFECTIVE2 (SRD2) in the model plant *Arabidopsis thaliana* is gametophytic lethal (Ohtani and Sugiyama 2005; Ohtani et al. 2008). Notably, a point mutation of *SRD2* in the *srd2-1* mutant causes temperature-sensitive defects in development, growth, and organ regeneration (Ohtani and Sugiyama 2005; Ohtani et al. 2008, 2010). Further study of this mutant revealed that UsnRNA level is an indicator of the competency for cell proliferation in plants (Ohtani et al. 2015; Takayanagi et al. 2022). Similar phenotypes are observed in other *Arabidopsis* splicing-related mutants, including *root initiation defective1*, whose nucleolus-localized RNA helicase presumably involved in UsnRNP biogenesis is defective (Ohtani et al. 2013). These observations suggest that the active regulation of splicing activity via UsnRNP biogenesis is crucial for regulating cellular activity and functionality in plants (Ohtani 2015, 2017, 2018).

Several studies have highlighted the importance of pre-mRNA splicing in the environmental responses of plants (Laloum et al. 2018; Kufel et al. 2022). Genome-wide transcriptome analyses have demonstrated that alternative splicing (AS) is actively regulated by changes in environmental conditions, such as light, temperature, nutrient levels, and pathogens (Reddy et al. 2013; Martin et al. 2021; Kufel et al. 2022; Enomoto et al. 2023). Calixto et al. (2018) performed extensive AS analyses under fluctuating temperature and light conditions and observed dynamic and rapid changes in selective splicing in response to these factors. A critical AS target identified by the authors was the *U2B"-LIKE* gene, encoding a subunit of U2snRNP; changes in temperature and daylength affected the amounts of specific splicing isoforms of *U2B"-LIKE* transcripts, while the *u2b"-like* mutant was unable to respond to environmental changes, resulting in growth failure (Calixto et al. 2018). Moreover, loss-of-function mutants of SERINE/ARGININE-RICH (SR) proteins such as SRp30 and SR45, which regulate AS by affecting the recruitment of spliceosomes to specific splice sites (Lorković et al. 2000), show abnormal abiotic stress responses (Carvalho et al. 2010; Filichkin et al. 2010). SKI-interacting protein (SKIP), an interactor of SR45, regulates most changes in AS induced by salt stress (Feng et al. 2015). These findings suggest that plants actively alter the variation and functionality of pre-mRNA splicing regulators in response to environmental stress, allowing them to reconfigure the splicing variants to fit the gene expression patterns required for particular cellular functions under specific environmental conditions. This view was supported by genome-wide comparative analysis of AS (Martin et al. 2021), which revealed a reduction in core spliceosomal activity upon exposure to abiotic stress.

Treatment of *Arabidopsis* with a pre-mRNA splicing inhibitor can mimic the molecular responses (i.e., gene expression patterns) observed under abiotic stress (AlShareef et al. 2017; Ling et al. 2017), raising the possibility that changes in splicing efficiency and/or the induction of AS can generate molecular signals that trigger the stress response in plants. Thus, plant cells might sense and reflect their stressed states via changes in AS.

In the present study, we investigated the effects of cellular stress-inducing chemicals on the AS of *SERINE-ARGININE PROTEIN30* (*SRp30*) (Lopato et al. 1999) and *U1-70 K* (Golovkin and Reddy 1996, 2003), both of which encode splicing factors, as well as *ASCORBATE PEROXIDASE3* (*APX3*), encoding an antioxidant enzyme (Narendra et al. 2006), and *FOLYLPOLYGLUTAMATE SYNTHASE3* (*FPGS3*), encoding a cytosolic enzyme that catalyzes folate polyglutamylation (Ravanel et al. 2001), in *Arabidopsis*. These four genes are known to undergo extensive regulation through AS (Ling et al. 2017). Different types of cellular stress induced different patterns of AS of *U1-70 K* and *FPGS3.* In addition, U1 snRNA levels changed in response to stress inducers, possibly leading to differential AS. These results provide insight into the molecular mechanisms underlying the induction of AS under different cellular stress conditions.

## Materials and methods

### Plant materials and growth conditions

*Arabidopsis thaliana* accession Columbia (Col-0) was used for treatment with inducers of cellular stress. The *Arabidopsis* accession Landsberg *erecta* (L*er*) and the *srd2-1* mutant (L*er* background; Yasutani et al. 1996) were used for mutant analysis. A series of *Arabidopsis* plants with variations in their snRNA levels were used for analysis, as described in Ohtani et al. (2015). The mutants were created by introducing *SRD2* promoter-driven *SRD2* homologous genes from rice, tobacco, poplar, *Physcomitrium patens*, and human into the *srd2-1* mutant background (Ohtani et al. 2015). Surface-sterilized seeds were sown on Murashige and Skoog medium that had been supplemented with 1.0% (w/v) sucrose (buffered to pH 5.7 with 0.05%, w/v, 2-morpholinoethanesulfonic acid monohydrate) and solidified with 1.5% (w/v) agar (Ohtani and Sugiyama 2005; Ohtani et al. 2008, 2010, 2013, 2015). The plates were incubated in the dark at 4 °C for 2 d, transferred to a growth chamber, and incubated at 22 °C under continuous light for 7 d.

### Treatment with cellular stress inducers

Seven-day-old seedlings were completely immersed in solutions of cellular stress inducers (concentrations indicated in Table S1) and incubated for 24 h in a growth chamber at 22 °C under continuous light (45–85 µmol m^−2^ s^−1^). The concentrations used for treatment were determined according to published reports describing the effects of each chemical in plants (Table S1).

### AS analysis using RT-PCR

Following treatment with inducers of cellular stress, the seedlings were collected, immediately frozen in liquid nitrogen, and stored at −80 °C. The samples were ground to a powder in liquid nitrogen, and total RNA was extracted using an RNeasy Mini Kit (Qiagen, Hilden, Germany), according to the manufacturer’s protocol. The RNA samples were treated with RQ1 RNase-free DNase (Promega, Madison, WI, USA), and 1 μg of the resulting RNA was used for a reverse transcription reaction with an oligo(dT)_12–18_ primer (Roche, Basel, Switzerland) in a 20-µL reaction volume using SuperScript III reverse transcriptase (Thermo Fisher Scientific, Waltham, MA, USA). The resulting solution was used as a template for the subsequent PCR, along with each gene-specific primer pair (Table S2) and Quick-Taq HS DyeMix (Toyobo, Osaka, Japan). The PCR conditions were 94 °C for 2 min, followed by 30 cycles (for *FPGS3* and *APX3*), 35 cycles (for *U1-70 K*), or 40 cycles (for *atSRp30*) of 94 °C for 30 s, 56 °C for 30 s, and 72 °C for 30 s, with a final extension at 72 °C for 7 min. The PCR products were resolved by electrophoresis in 2.5% (w/v) Agarose S (Toyobo) gels. After staining the gels with SYBR Gold (Thermo Fisher Scientific), gel images were analyzed using a FastGene FAS-V Gel Imaging System (NIPPON Genetics, Tokyo, Japan).

For sequencing analysis of the RT-PCR products, amplified DNA fragments were separated by gel electrophoresis, cut out from the agarose gel, cloned into the pGEM T-Easy vector (Promega), and subjected to DNA sequencing using an Applied Biosystems BigDye Terminator v3.1 Cycle Sequencing kit (Thermo Fisher Scientific) and an Applied Biosystems 3130xl Genetic Analyzer (Thermo Fisher Scientific).

### Prediction of protein sequence and functional domains

Sequencing results of the AS isoforms were analyzed using GENETYX v. 14 software (GENETYX, Tokyo, Japan). A diagram of the protein structure predicted from each AS isoform was generated using A Plasmid Editor (ApE) software v. 2.0.49.0 (Davis and Jorgensen 2022). The results were compared with those for the functional isoforms of each protein in UniProt (https://www.uniprot.org/) to determine the presence or absence of functional domains in the proteins.

### RT-qPCR analysis of UsnRNAs

RT-qPCR analysis was performed according to Ohtani et al. (2015). Briefly, first-strand cDNA was used as the template, and UsnRNA levels were measured by qPCR using the LightCycler 96 system with LightCycler 480 SYBR Green I Master (Roche). *18S rRNA* was used as an internal control. Primers targeting all variants of each UsnRNA used for RT-qPCR were described by Ohtani et al. (2015), and shown in Table S2. For statistical analysis, Student’s *t*-test was performed between the inducer-treated and mock-treated samples.

## Results

### Inducers of cellular stress affect the AS of *SRp30*, *U1-70 K*, *APX3*, and *FPGS3*

**Fig. 1:**
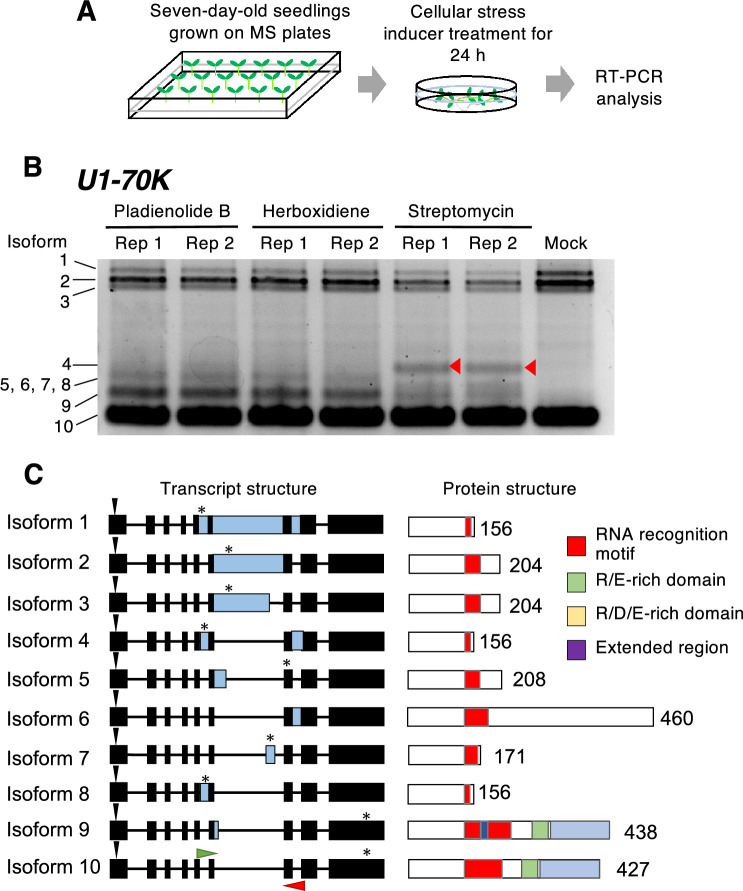
Alternative splicing (AS) patterns induced by cellular stress. **A** Seven-day-old wild-type (Col-0) seedlings were treated with inducers of cellular stress for 24 h and subjected to RT-PCR analysis. **B** Two replicates (Rep) of RT-PCR analysis of *U1-70 K* expression following treatment with different cellular stress inducers. Ten AS isoform bands were detected. Red arrowheads indicate streptomycin-specific AS bands. **C** Transcript structure and protein structure predicted from the PCR products of the 10 AS isoform bands shown in **B**. The exon and intron structures of each AS isoform are shown. Isoform 10 is expected to produce a functional U1-70 K protein. Exons are indicated by black boxes, exonized regions to compare with the isoform 10 are indicated by light blue boxes, introns are indicated by black lines, and the positions of start and stop codons are indicated by black arrowheads and asterisks, respectively. Green and red horizontal arrowheads indicate the position of the forward and reverse primers used for RT-PCR analysis, respectively. The predicted functional domains of the protein generated from each AS isoform are shown on the right. Numbers indicate the number of amino acid residues in each protein isoform. (Color figure online)

**Fig. 2:**
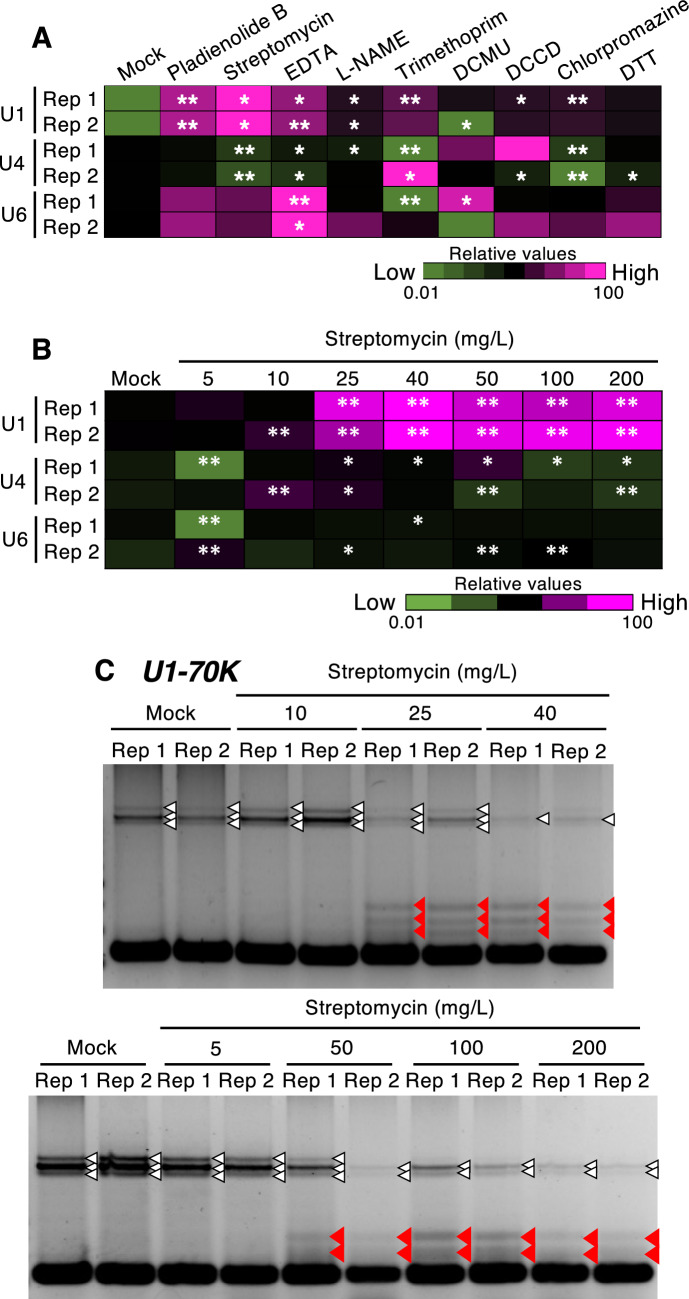
Changes in UsnRNA abundance and AS patterns induced by cellular stress. **A** Seven-day-old wild-type (Col-0) seedlings were treated with inducers of cellular stress for 24 h and subjected to RT-qPCR analysis of U1 snRNA, U4 snRNA, and U6 snRNA levels. *18S rRNA* was used as an internal standard. The results are shown as relative expression values compared to the standardized expression values for each UsnRNA (mean values of three biological replicates). Asterisks indicate significant differences compared with the Mock treatment (*n* = 3, *p < 0.05, **p < 0.01; Student’s* t*-test with Bonferroni correction). **B** Seven-day-old wild-type (Col-0) seedlings were treated with 0 (Mock), 5, 10, 25, 40, 50, 100, or 200 mg/L streptomycin for 24 h and subjected to RT-qPCR analysis of U1 snRNA, U4 snRNA, and U6 snRNA levels. *18S rRNA* was used as an internal standard. The results are shown as relative values. Asterisks indicate significant differences compared with the Mock treatment (*n* = 3, *p < 0.05, **p < 0.01; Student’s *t*-test with Bonferroni correction). **C** Two replicates (Rep) of RT-PCR analysis of *U1-70 K* expression following streptomycin treatment. White and filled red triangles indicate AS bands detected in the Mock-treated and streptomycin-treated samples, respectively. (Color figure online)

We first tested the effects of known 31 inducers of cellular stress on regulation by AS. For this purpose, we selected commercially available chemicals that were reported to disturb cellular activity in plants (Table S1). We soaked seven-day-old wild-type *Arabidopsis* seedlings in solutions of these chemicals for 24 h at 22 °C (Fig. 1A) and performed reverse transcription (RT)-PCR analysis of the expression of *SRp30* and *U1-70 K*, encoding splicing factors (Lopato et al. 1999; Golovkin and Reddy 1996, 2003); *APX3*, encoding microsomal ascorbate peroxidase (Narendra et al. 2006); and *FPGS3*, encoding cytosolic folylpolyglutamate synthase (Ravanel et al. 2001) in these seedlings. The *APX3* and *FPGS3* genes have been well documented to be alternatively spliced (Shigeoka et al. 2002; Ling et al. 2017; Calixto et al. 2018). We used pladienolide B and herboxidiene, well-known pre-mRNA splicing inhibitors (AlShareef et al. 2017; Ling et al. 2017), as positive controls for AS (Figs. 1 and 2; Table S1; Figs. S1 and S2). Both splicing inhibitors altered the splicing patterns of all genes examined (Fig. 1B; Figs. S1 and S2), producing similar patterns of splicing, which is consistent with the finding that both splicing inhibitors interact with the U2 snRNP subunit SF3b (AlShareef et al. 2017; Ling et al. 2017).

Table 1 shows a summary of 14 inducers of cellular stress that affected the AS of *SRp30*, *U1-70 K*, *APX3*, and *FPGS3* among 31 tested chemicals in Table S1. The other 17 chemicals did not show stable effects on their AS patterns. These findings indicate that the inhibition of specific cellular activity can strongly affect regulation through AS; specifically, disrupting nitric oxide metabolism, plastid/mitochondria function, the biosynthesis of amino acids and nucleic acids, cell cycle regulation, and histone modification could modulate AS (Table 1). Consistent with the previous finding that inhibiting plastid function can affect AS (Petrillo et al. 2014), we determined that streptomycin, an inhibitor of eubacterial ribosome function, strongly affected the splicing patterns of all genes examined (Fig. 1B; Figs. S1 and S2).

**Table 1:** Summary of effects of chemical on alternative splicing

Chemical^a^	Known Effects^b^	*atSRp30*	*U1-70 K*	*APX3*	*FPGS3*
GSNO	Source of bioavailable NO	−	−	−	Affected
L-NAME	NO synthase inhibitor	−	−	−	Affected
Potassium nitrate	ATPase inhibitor	−	−	−	Affected
Trimethoprim	Folate synthesis inhibitor	−	−	−	Affected
Glyphosate	Shikimate pathway inhibitor	−	−	−	Affected
Cdk4/6 Inhibitor IV	CDK inhibitor	−	−	−	Affected
Caffeic Acid	GST inhibitor	−	−	−	Affected
Trichostatin A	HDAC inhibitor	−	−	−	Affected
5-Flurouracil	DNA and RNA synthesis inhibitor	−	−	−	Affected
Hydroxyurea	DNA replication inhibitor	−	−	−	Affected
EDTA	Chelator	−	−	Affected	−
Streptomycin	Eubacterial ribosome inhibitor	Affected	Affected	Affected	Affected
Pladienolide B	Splicing inhibitor	Affected	Affected	Affected	Affected
Herboxidiene	Splicing inhibitor	Affected	Affected	Affected	Affected

^a^*GSNO* *S*-Nitrosoglutathione, *L-NAME* NG-Nitro-L-arginine methyl ester hydrochloride, *EDTA* Ethylenediaminetetraacetic acid. ^b^*NO* Nitric noxide, *CDK* Cyclin-dependent kinase, *GST* Glutathione *S*-transferase, *HDAC* Histone deacetylase

### Characterization of AS patterns of *FPGS3* and *U1-70 K*

Inducers of cellular stress had different effects on the splicing patterns of different genes (Fig. 1B; Figs. S1 and S2; Table 1). Among the four genes examined, the most sensitive to cellular stress in terms of AS was *FPGS3*, with its splicing pattern influenced by 13 inducers of cellular stress (Fig. S2; Table 1). Each inducer of cellular stress generated different levels of *FPGS3* AS isoforms (Fig. S2), suggesting that differential AS mechanisms could be activated by each stressor. Moreover, in addition to pladienolide B and herboxidiene, only streptomycin disturbed the splicing of all four genes (Fig. 1B; Figs. S1 and S2).

Sanger sequencing analysis successfully detected 10 and 18 isoforms for *U1-70 K* and *FPGS3*, respectively (Fig. 1B; Fig. S2). Most isoforms were derived from variations in intron retention, alternative 5′/3′ splice site selection, or a combination of the two (Fig. 1B; Fig. S2). Analysis of splicing efficiency scores, which indicate the sequence similarity of a 5′/3′ splice site compared with the consensus sequence (5′ splice site: (C/A)AGGGURAGU; 3′ splice site YnNYAGG) (Breathnach and Chambon 1981), revealed that the AS sites had splicing efficiencies comparable to, or higher than, those of the splicing isoform producing a functional protein (Fig. S3).

Streptomycin treatment altered the splicing of *U1-70 K* and *FPGS3* in a manner unlike that of the splicing inhibitors pladienolide B and herboxidiene (Fig. 1B and Fig. S2). Streptomycin induced the formation of isoform 4 of *U1-70 K* (indicated by a filled red triangle in Fig. 1B), while the splicing inhibitors induced the formation of isoform 9. In the case of *FPGS3*, isoform 1 strongly accumulated following streptomycin treatment, while the splicing inhibitors induced the formation of all types of AS isoforms (Fig. S2). In both cases, the isoforms induced by streptomycin treatment would produce truncated proteins lacking important functional domains (Fig. 1C; Fig. S2). These findings suggest that the effects of streptomycin on *U1-70 K* and *FPGS3* splicing are mechanistically distinct from those of the U2B”-targeting splicing inhibitors and that streptomycin negatively regulates *U1-70 K* and *FPGS3* expression.

### Streptomycin-induced increases in U1 snRNA levels may influence AS

Active changes in UsnRNA levels can be induced by stress (Younis et al. 2013; Ohtani et al. 2015). Therefore, we examined the effects of treatment with inducers of cellular stress on UsnRNA levels using quantitative RT-PCR (RT-qPCR). UsnRNA abundance was significantly altered by the stress inducers, with more pronounced effects caused by those that could effectively induce AS, particularly pladienolide B and streptomycin, for which the amount of U1 snRNA was greater than that under mock treatment (Fig. 2A). U4 and U6 snRNA abundance increased and/or decreased in response to several stress inducers, but these effects were not reproducible (Fig. 2A).

**Fig. 3:**
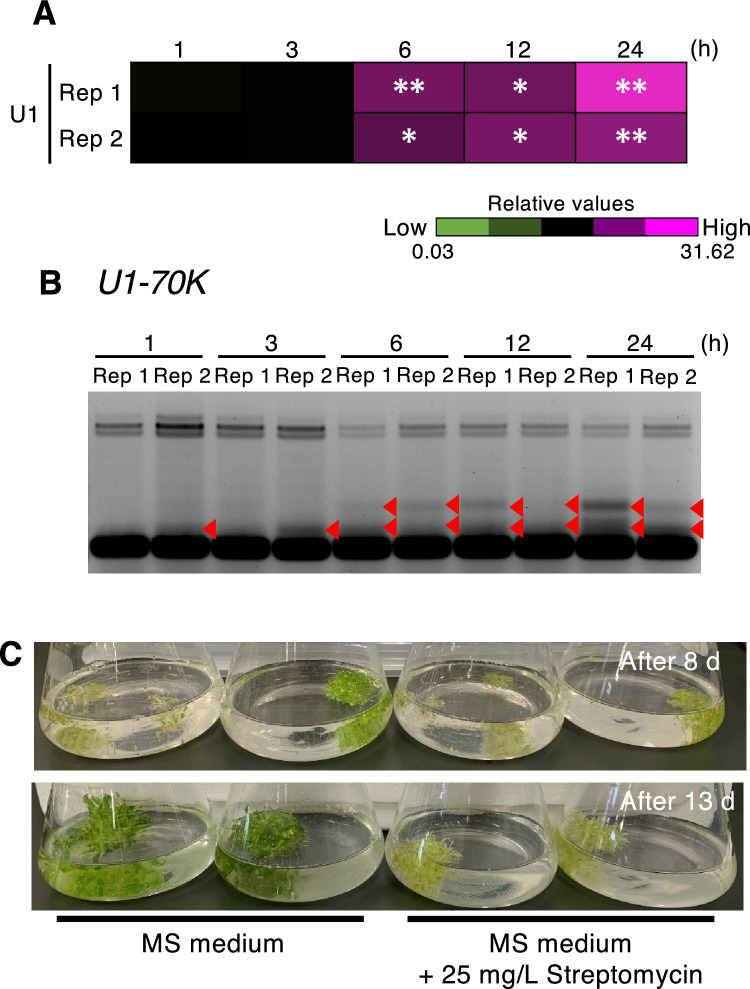
Streptomycin-dependent changes in *U1-70 K* AS patterns, U1 snRNA levels and seedling growth. **A, B** Treatment time-dependent changes in U1 snRNA levels (**A**) and *U1-70 K* AS patterns (**B**) in response to streptomycin treatment. Seven-day-old wild-type (Col-0) seedlings were treated with 25 mg/L streptomycin for 1, 3, 6, 12, or 24 h and subjected to RT-qPCR analysis. In **A**, the results are shown as relative values (average of three biological replicates). Asterisks indicate significant differences compared with the sample treated for 1 h (n = 3, *p < 0.05, **p < 0.01; Student’s *t*-test with Bonferroni correction). Filled red triangles in **B** indicate the AS bands detected in the streptomycin-treated samples. **C** Wild-type seedlings were treated with 25 mg/L streptomycin for 24 h and grown on MS medium without or with 25 mg/L streptomycin for an additional 8 d (upper panel) or 13 d (lower panel). (Color figure online)

We tested the dose-dependent effects of streptomycin using concentrations up to 200 mg/L. Both increased U1 snRNA abundance and aberrant splicing patterns of *U1-70 K* appeared in plants treated with ≥ 25 mg/L of streptomycin (Fig. 2). Increased concentrations of streptomycin also decreased *U1-70 K* expression (Fig. 2C). In addition, analysis of the effects of streptomycin treatment for 1, 3, 6, 12, and 24 h revealed that abnormalities in U1 snRNA abundance and splicing patterns could be detected after 6 h of treatment (Fig. 3A, B). Streptomycin treatment always simultaneously triggered an increase in U1 snRNA levels and abnormal regulation of splicing; thus, streptomycin-induced AS is likely to be attributed to changes in U1 snRNA levels.

Treatment with 25 mg/L streptomycin inhibited seedling greening and subsequent seedling growth (Fig. 3C). We monitored the recoverability of this streptomycin-derived inhibition of growth by transferring seedlings treated with 25 mg/L streptomycin for 24 h to medium lacking streptomycin (or containing 25 mg/L streptomycin as a control; Fig. 3C). After 8 d of culture, obvious differences in greening were not apparent in the presence or absence of streptomycin; however, after an additional 5 d of culture, plants grown in medium lacking streptomycin had turned green and grown larger than those in control medium containing streptomycin (Fig. 3C). These findings indicate that 25 mg/L streptomycin is not lethal for seedlings, but is instead a recoverable stress condition, providing evidence that stress (streptomycin) induces AS, which influences plant development and physiology.

### Defective snRNA transcriptional activity affects AS

**Fig. 4:**
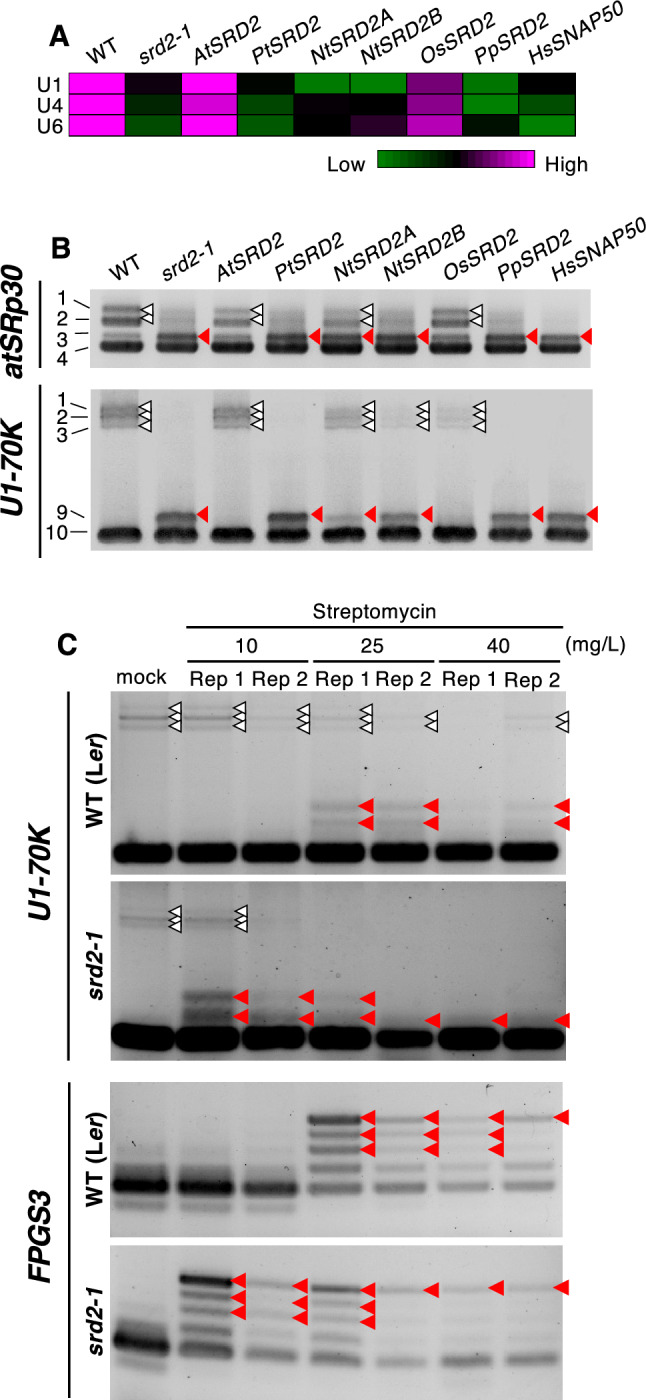
Effects of the *srd2-1* mutation on UsnRNA abundance and AS patterns. **A** Seedlings of the wild type (WT), *srd2-1*, and *srd2-1* carrying *SRD2* homologs from *Arabidopsis thaliana* (*At*), *Populus trichocarpa* (*Pt*), *Nicotiana tabacum* (*Nt*), *Oryza sativa* (*Os*), *Physcomitrium patens* (*Pp*), or human (*HsSNAP50*) were grown at 28 °C and subjected to RT-qPCR analysis to quantify UsnRNA levels. The results are shown as relative values (average values of three biological replicates). **B** Typical results of RT-PCR analyses of *atSRp30* and *U1-70 K* expression. White and filled red triangles indicate AS bands detected in Mock-treated and streptomycin-treated samples, respectively. **C** Seven-day-old wild-type (L*er*) and *srd2-1* seedlings were treated with 0 (Mock), 10, 25, or 40 mg/L streptomycin and subjected to RT-PCR analysis. Typical results of two replicates (Rep 1 and Rep 2) for *U1-70 K* and *FPGS3* are shown. White and red triangles indicate AS bands detected in Mock-treated and streptomycin-treated samples, respectively. (Color figure online)

The finding that inducers of cellular stress affect UsnRNA levels (Figs. 2 and 3) prompted several questions, including whether this effect was mediated by the transcriptional regulation of UsnRNAs. We therefore examined the AS patterns of *atSRp30* and *U1-70 K* in a series of *Arabidopsis* plants with variations in snRNA levels, which were created by introducing *SRD2* homologs from other species into the snRNA transcription–defective mutant *srd2-1* (Ohtani et al. 2015). As *srd2-1* is a high temperature-sensitive mutant, we grew the plants at the restrictive temperature of 28 °C for 12 d. Analysis of the relative amounts of U1, U4, and U6 snRNAs indicated that the levels of all UsnRNAs were significantly lower in *srd2-1* than in the wild type but were restored to wild-type levels by introducing *AtSRD2* or the rice (*Oryza sativa*) homolog *OsSRD2* into the mutant background (Fig. 4A; Ohtani et al. 2015). In other lines, UsnRNA levels were partially elevated in some cases, although they did not fully recover to wild-type levels (Fig. 4A; Ohtani et al. 2015). RT-PCR analysis revealed abnormal splicing patterns of *atSRp30* and *U1-70 K* in *srd2-1* (Fig. 4B), with fewer longer isoforms (i.e., isoforms 1 and 2 of *atSRp30* and isoforms 1 to 3 of *U1-70 K*) and increased levels of specific isoforms (i.e., isoform 3 of *atSRp30* and isoform 9 of *U1-70 K*; Fig. 4B). These findings demonstrate the importance of UsnRNA transcription for the proper regulation of AS.

To test the possibility that the abnormal AS patterns caused by treatment with stress inducers are mediated by the abnormal regulation of UsnRNA transcription, we examined the sensitivity of *srd2-1* to streptomycin (Fig. 4C). The AS patterns of *U1-70 K* and *FPGS3* in wild-type seedlings were altered by treatment with streptomycin at concentrations over 25 mg/L (Fig. 4C). However, in the *srd2-1* mutant, abnormal AS patterns were detected following treatment with 10 mg/L streptomycin (Fig. 4C), indicating that this mutant has increased sensitivity to streptomycin. Taken together, these findings indicate that the transcriptional regulation of snRNA is a key mechanism driving cellular stress–induced AS.

## Discussion

###  Plants transduce cellular stress signals into changes in gene expression via the regulation of AS

The inhibition of certain cellular functions, such as nitric oxide metabolism, ATP metabolism, plastid and/or mitochondria function, and genome stability, can result in abnormal AS patterns (Table 1; Fig. 1: Figs. S1 and S2). The regulation of AS can be understood by examining mRNA turnover dynamics, including transcription and degradation. Among the genes tested in this study, *atSRp30*, *U1-70 K*, and *FPGS3* are known to be upregulated by heat, drought, DNA damage, and oxidative stress (Chen et al. 2020; Kilian et al. 2007). In addition, an analysis of mRNA half-life using *Arabidopsis* seedlings treated with actinomycin D indicated that the mRNA half-lives of *atSRp30*, *U1-70 K, FPGS3*, and *APX3* are 9.873 h, 3.645 h, 3.315 h, and 9.278 h, respectively, under normal conditions (Narsai et al. 2007). Here, we detected prominent changes in AS of *U1-70 K* after 6 h of streptomycin treatment (Fig. 3), suggesting that newly biosynthesized pre-mRNAs undergo streptomycin-specific regulation of AS. Notably, the effects on the AS of these stress-responsive genes differed with the use of different inducers of cellular stress and from gene to gene (Fig. 1; Figs. S1 and S2). Thus, different inducers of cellular stress generate different AS variations, which may function to fine-tune gene expression in response to the environment.

In the present study, we identified streptomycin as a stress inducer that affected the AS of all four genes examined (Table 1; Fig. 1; Figs. S1 and S2). Streptomycin is a fungal-type inhibitor of ribosomal function that restricts mitochondrial and chloroplast function in plant cells. A previous study revealed that retrograde signals coming from chloroplasts may regulate AS in the nucleus (Petrillo et al. 2014). Building on these findings, our results suggest that chloroplast activity is tightly linked with the regulation of AS in plant cells. Environmental factors, such as light, temperature, and daylength, can directly affect photosynthetic activity. Therefore, environmental changes would be recognized by the cell as changes in photosynthetic activity, which can lead to the regulation of AS.

### AS is regulated by UsnRNA dynamics in response to cellular stress

We also showed that UsnRNA levels are crucial for the cellular stress–dependent regulation of AS (Figs. 2, 3, 4). The *Arabidopsis srd2-1* mutant, with defective snRNA transcription (Ohtani and Sugiyama 2005), showed abnormal AS patterns at the restrictive temperature (Fig. 4A, B) and increased sensitivity to streptomycin in terms of AS abnormalities (Fig. 4C), indicating that streptomycin-induced abnormalities in AS are mediated by SRD2-dependent transcriptional regulation of UsnRNA. Currently, no direct evidence explains how streptomycin affects snRNA transcription in plants. However, a previous study reported that in human liver cells treated with both penicillin and streptomycin, a wide range of genes were influenced in terms of expression and histone modifications, including insulin signaling factors and transcription factors (Ryu et al. 2017). This suggests that streptomycin may also impact the expression of snRNA transcriptional regulators, potentially altering snRNA transcription in plant cells. The upstream factors of SRD2 remain unknown. Therefore, uncovering the molecular link between streptomycin and SRD2-dependent regulation of UsnRNA transcription will be an important challenge for future research.

Notably, inducers of cellular stress increased U1 snRNA levels (Figs. 2 and 3). UsnRNAs are core factors of the molecular spliceosome machine; thus, changes in UsnRNA levels are likely to directly affect splicing regulation. As an example, an increase in the levels of U1 snRNAs (U1 snRNPs) can result in the recruitment of U1 snRNPs to splice sites that are not ordinarily used (Fig. S4). Or, changes in which U1 snRNA species are transcribed may also affect the preference of splice sites to be targeted (Fig. S4). As supporting this idea, it is also known that the components of U1 snRNPs, such as U1A, U1-70 K, LETHAL UNLESS CBC7 (LUNC7), PRP39A and PRP40A, are transcriptionally upregulated in response to stresses (Kilian et al. 2007; de Francisco Amorim et al. 2018; Chen et al. 2020). Moreover, each UsnRNA species has a different level of stress responsiveness (Fig. 2), leading to the disturbed balance among the UsnRNAs and the inhibition of splicing events. U1 snRNA is known to be present in larger amounts than other UsnRNAs and functions in the regulation of nascent mRNA biosynthesis (i.e., transcription) (Kaida et al. 2010; Berg et al. 2012), suggesting that increased U1 snRNA levels might also affect transcriptional activity under stress conditions.

We propose a model in which the inhibition of specific cellular activities, such as chloroplast function, can affect AS patterns via changes in UsnRNA levels (Fig. S4). This proposed mechanism is believed to represent one way in which immobile plants continuously sense changes in their environment and reflect them in their transcriptomes. Additional studies of the stress responsiveness of UsnRNP activity should further elucidate the roles of pre-mRNA splicing in the environmental responses of plants.

## Supplementary Information

Below is the link to the electronic supplementary material.

Supplementary file1 (PDF 216 KB)

Supplementary file2 (XLSX 25 KB)

## Data Availability

Not applicable
