# Supplementary material for: Chemically-induced cellular stress signals are transmitted to alternative splicing via UsnRNA levels to alter gene expression in Arabidopsis thaliana
Source: Plant Mol Biol. 2025 Mar 16;115(2):46. doi: 10.1007/s11103-025-01575-9 (PMC11911268; doi:10.1007/s11103-025-01575-9)
Supplement: Supplementary file 1 — Supplementary file1 (PDF 216 KB) [file 11103_2025_1575_MOESM1_ESM.pdf]

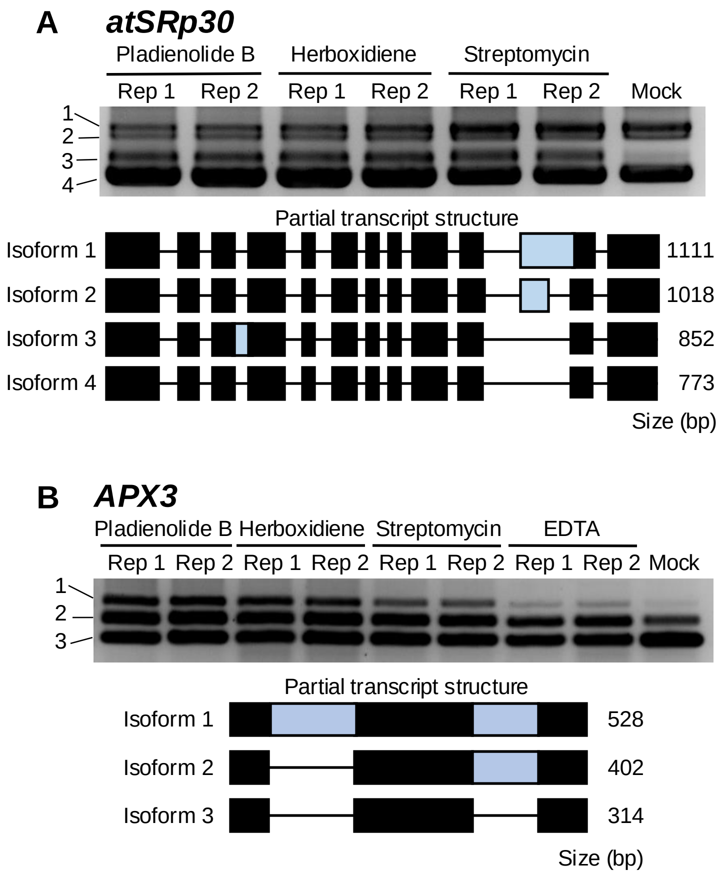

Fig. S1. AS patterns of *atSRp30* and *APX3* induced by cellular stress.

Seven-day-old wild-type (Col-0) seedlings were treated with inducers of cellular stress for 24 h and subjected to RT-PCR analysis. Two replicates (Rep) of the RT-PCR analysis are shown. Partial transcript structures are presented for each AS isoform, showing exons (boxes) and introns (lines). Black boxes: exons found in isoforms producing functional proteins. Light blue boxes: exonized regions to compare with the isoforms expected to produce functional proteins. **A** *atSRp30*; isoform 4 is expected to produce a functional atSRp30 protein. **B** *APX3*; isoform 3 is expected to produce a functional APX3 proteins.

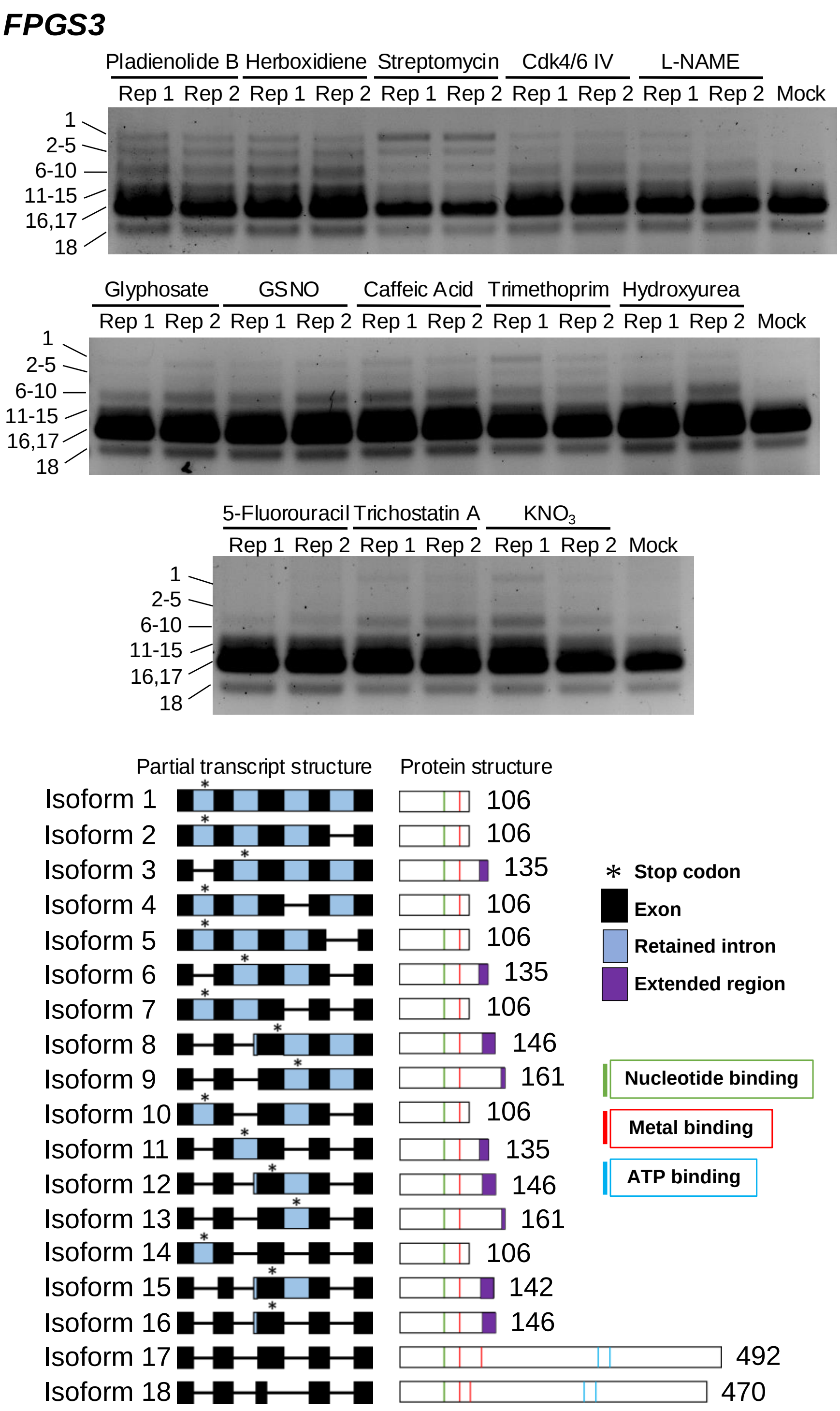

Fig. S2. AS patterns of *FPGS3* induced by cellular stress.
Seven-day-old wild-type (Col-0) seedlings were treated with inducers of cellular stress for 24 h and subjected to RT-PCR analysis of *FPGS3* expression. Two replicates (Rep) of the RT-PCR results are shown, and 18 AS isoforms were detected. Partial transcript structures are presented for each AS isoform, showing exons (boxes) and introns (lines), and expected full-length protein structures are shown. Isoform 17 is expected to produce a functional FPGS3 protein. Black boxes: exons found in isoforms producing functional proteins. Light blue boxes: exonized regions to compare with the isoform 17. Numbers indicate the number of constituent amino acid residues for each isoform protein.

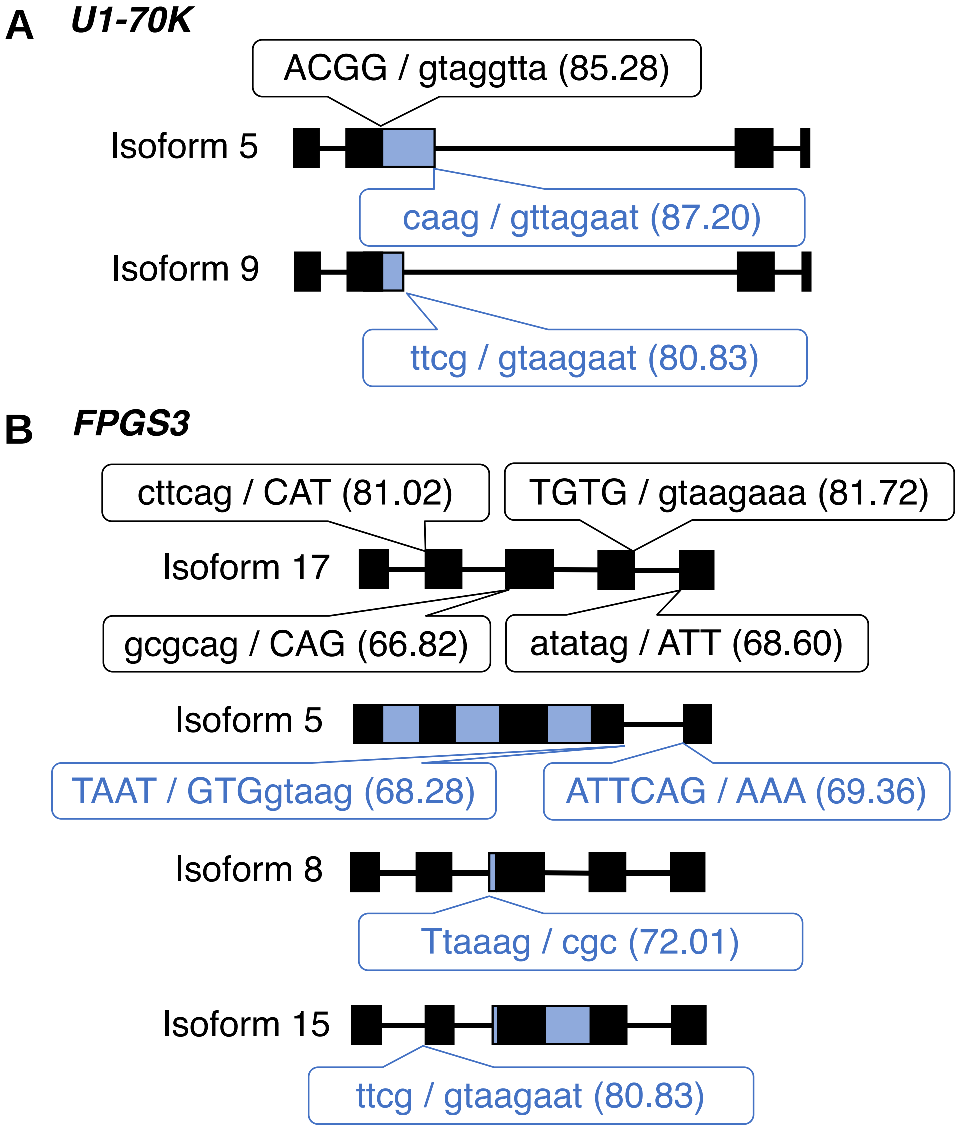

Fig. S3. Splicing efficiency scores of alternative 5′ and 3′ splice sites in *U1-70K* and *FPGS3*.
Alternative 5′ and 3′ splice sites and their splicing efficiency scores in the **A** *U1-70K* and **B** *FPGS3* AS isoforms. Splicing efficiency scores (%) represent the percentage of similarity to the consensus splice site sequence, as calculated using GENETYX software. Uppercase and lowercase letters indicate sequences corresponding to exons and introns, respectively.

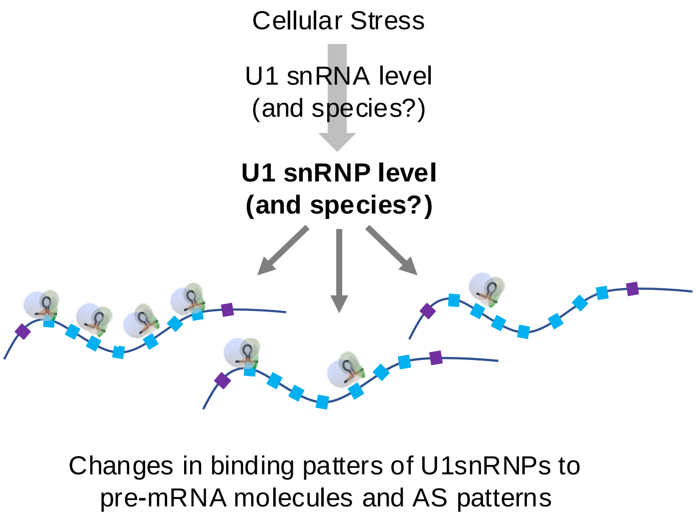

Fig. S4. Model of the regulatory mechanisms of AS in response to cellular stress. Cellular stress can alter the amount of U1 snRNA, leading to changes in U1 snRNP levels. U1 snRNA species are also possibly changed. These differential U1 snRNP levels (and/or species) in response to stress influence their recognition of potential splice sites, which can alter the complexity of AS patterns.
